# Inappropriate use of antibiotics among communities of Gondar town, Ethiopia: a threat to the development of antimicrobial resistance

**DOI:** 10.1186/s13756-017-0272-2

**Published:** 2017-11-07

**Authors:** Daniel Asfaw Erku, Abebe Basazn Mekuria, Sewunet Admasu Belachew

**Affiliations:** 1 Department of Clinical Pharmacy, School of Pharmacy, University of Gondar Chechela Street, Lideta Sub city Kebele, 16 Gondar, Ethiopia; 2 Department of Pharmacology, School of Pharmacy, University of Gondar Chechela Street, Lideta Sub city Kebele, 16 Gondar, Ethiopia; 3 Department of clinical pharmacy, School of Pharmacy, College of Medicine and Health Sciences, University of Gondar, P.O. Box: 196, Gondar, Ethiopia

**Keywords:** Inappropriate use, Antimicrobials, Drug resistance, Community, Ethiopia

## Abstract

**Background:**

The emergence of antimicrobial resistance, the main cause of morbidity and mortality from otherwise treatable infections, is largely attributed to the inappropriate use of antimicrobials. However, data on the extent of inappropriate use of antibiotics in the community is scarce in Ethiopia. The aim of present study is to document the extent of inappropriate use of antibiotics and its associated factors among the communities of Gondar, Northwest Ethiopia.

**Methods:**

A community based cross-sectional survey was conducted on a total of 650 participants in Gondar town, northwest Ethiopia from December 1, 2016 to January 30, 2017. Descriptive statistics, univariate and multivariate logistic regression analysis were also performed to express different variables and to examine factors associated with inappropriate use of antibiotics.

**Results:**

According to the finding of our study, 315 (48.5%) of the participants took antibiotics in the past 1 year, of which 115 (35.9%) of them used inappropriately. Amoxicillin (72%) was the most commonly utilized antibiotics and respiratory tract infection (40.9%) was the most common disease condition to which antibiotics had been sought. About 36.8% of the respondents got antibiotics from community drug retail outlets without a prescription and 67.9% of respondents had discontinued the use of antibiotics once their symptoms subside. Low educational status (AOR = 5.01, 95% CI = 2.62–9.34), being employed (AOR = 2.12, 95% CI = 1.81–7.29) and unsatisfied with health care services provided (AOR = 5.41, 95% CI = 2.71-14.21) were found to be strong predictors of inappropriate use of antibiotics use among the community.

**Conclusion:**

Inappropriate use of antibiotics was found to be considerably high in the communities of Gondar, northwest Ethiopia. Taking into consideration the heightened importance of comprehensive knowledge in the rational use of antibiotics, different stakeholders working in the public health sectors should provide a comprehensive and customized education to the public so as to improve their knowledge about antibiotics. It is also essential to adopt a strong and explicit line of actions towards the accessibility of antibiotics without a valid prescription in community medicine retail outlets.

## Background

The emergence of antimicrobial resistance (AMR), the main cause of morbidity and mortality from otherwise treatable infections, is largely attributed to the use, over use or misuse of antimicrobials [1]. The development of AMR coupled with the downturn in the development of new antimicrobials in the pharmaceutical industry creates unexpected challenges in the effective management of infections [2]. Annually, multi-drug resistant (MDR) bacteria is estimated to claim the lives of more than 20,000 patients in North America, 25,000 patients in Europe and more than 90,000 patients in Southern Asia [3, 4].

A number of researchers underlined the relationship between the inappropriate use of antimicrobials in the community and the emergence of antimicrobial resistance [5]. According to WHO (World Health Organization), more than two third of all antibiotics are used in the community, of which about 30% is used inappropriately [2]. Due to this, interventions focusing on the in the community such as improving access to medical services, reducing unnecessary use of antibiotics, taking a full course of treatment, and not sharing medications with other people are recommended [6]. Many previous studies documented factors associated with inappropriate use of antibiotics including culture [7], educational status [8], residency [9], marital status [8], age [10], health insurance [8] and level of satisfaction with the health care services [11, 12], and storing antibiotics at home [13].

In Ethiopia, there are signs of irrational use of antibiotics by the community, patients as well as by health care providers. According to the baseline survey conducted by Food, Medicine and Healthcare Administration and Control Authority of Ethiopia (FMHACA), about two third of patients (70%) patients who visited outpatient clinics have had one or more antibiotics prescribed with a percentage of irrational prescribing close to 40% [14]. A number of studies underlined the role of the general public in the emergence and spread of antibiotic resistance [15–18]. According to WHO, improving public access to medical facilities, reducing unnecessary and irrational use of antimicrobials, taking prescribed antimicrobials to their full course of therapy and not sharing medication with other people are some of the key issues of the general public in the fight against antimicrobial resistance [19]. However, the extent of inappropriate use of antibiotics in the urban and rural community settings has not yet been fully explored in Ethiopia. The present study aimed to document inappropriate use of antibiotics and its associated factors among the communities of Gondar, Northwest Ethiopia.

## Methods

### Study design and setting

A community based cross-sectional survey was conducted from December 1, 2016 to January 30, 2017 to determine inappropriate use of antibiotic among the communities Gondar town, northwest Ethiopia. We collected data between December 2016 and January 2017 as this season is associated with higher cases of infectious disorders and other common parasitic infections in Ethiopia. This study was approved by the ethical committee of School of Pharmacy, University of Gondar with an approval number of UoG-SoP-123/2016. Written informed consent from the participants was also obtained before conducting this study. Participants’ information obtained was kept confidential.

### Population and sampling

Gondar town, the study area, is found in Amhara regional state and is located 750 km Northwest of Addis Ababa (the capital city of Ethiopia). According the recent population and housing census report, Gondar town has a total of 207, 000 population [20]. The town has a total of 12 administrative zones (areas),1 referral hospital, and 1 defense hospital and 5 health centers. Single population proportion formula was used with the assumption of 95% confidence interval, 5% margin of error, the prevalence (p) of inappropriate use of antibiotics (30.9%) [21] and 5% for possible non-response to determine a final sample size of 720. Multistage stratified random sampling technique was used to select households in administrative areas (kebeles). Five administrative areas were selected randomly to get a representative sample. The number of households to be interviewed in each administrative area was determined in proportion with the total number of households found in each kebeles. A systematic random sampling method was then used to select the study participants. Lottery method was used to select a respondent whenever more than one eligible respondent found in the selected household.

### Survey instrument

Data collection was performed by five well trained final year pharmacy students through interviewer-administered questionnaire. The tool was created by modifying items in a previously used instrument regarding knowledge and use of antibiotics in the community [21], and items were thoroughly reviewed for relevance by a team of experts including experienced clinical pharmacists and public health experts. The survey instrument was further pre-tested on 45 households who were not included in the final analysis and relevant modifications were instituted before the commencement of actual data collection. The questionnaire asks respondents about the socio-demographic characteristics and knowledge as well as the use of antibiotics. Investigators took different antibiotics (in all dosage forms) with them to show participants whether they know and/or use the antibiotics in the last 1 year. In our study, inappropriate use of antibiotics is defined as the non-prescription use of antibiotics for themselves and/or their family members, or the use of leftover antibiotics or the use of prescribed antibiotics for a reason other than its intended for [22].

### Statistical analysis

All the statistical analyses were done using Social Sciences (SPSS) software version 21.0 for Windows (SPSS Inc., Chicago, IL). Frequencies and percentages were used to express different variables. Uivariate and multivariable logistic regressions were used to come up with predictors of inappropriate use of antibiotics. Associations with significance level of less than 0.20 (*p* < 0.20) in the univariate analysis were included in the multivariate logistic regression analysis. The results were adjusted for patients’ demographic and disease characteristics. OR with 95% CI were also computed along with corresponding *p*-value (*p* < 0.05) as cut off points for determining statistical significance.

## Results

Out of 720 households approached, 650 of them give consent and included in the study giving a response rate of 90.3%. The mean age of participants was 33.19 years with a standard deviation of ± 10.82. The majority of respondents were females (74.9%) and the mean family size was 4.2 with a standard deviation of ± 2.32. Details of socio-demographic characteristics of study participants are shown in Table 1.

**Table 1:** Socio-demographic characteristics and factors associated with inappropriate antibiotic among study participants, Gondar, Ethiopia 2017 (*N* = 650)

Variables	Total (*n* = 650)	Inappropriate use		AOR (95%CI)
		No (*n* = 200)	Yes (*n* = 115)	
Gender				
Male	163(25.9%)	127	36	–
Female	487 (74.9%)	408	79	–
Age				
< 29	132 (20.3%)	108	24	1.02 (0.38–1.92)
30–39	247 (38%))	212	35	1.13 (0.46–2.02)
40–49	123 (18.9)	98	25	1.01 (0.30–1.41)
50-59	89 (13.7%)	70	19	0.98 (0.27–1.71)
> 60	59 (9.1%)	47	12	1
Number of family size				
1–2	156 (24%)	121	35	–
3–5	383 (58.9%)	339	44	–
> 5	111 (17.1)	75	36	–
Family monthly income (in USD)				
< 100	331 (50.9%)	282	49	–
101–150	201 (30.9%)	170	31	–
> 150	118 (18.2%)	83	35	–
Education status				
Unable to read and write	182 (28%)	125	57	5.01 (2.62–9.34)
Primary education	201 (30.9%)	180	21	2.81 (1.32–6.146)
Secondary education	179 (27.5%)	159	20	1.96 (0.91–4.51)
Tertiary (college) education	88 (13.5%)	71	17	1
Employment status				
Unemployed	253 (38.9%)	209	44	1
Employed	397 (61.1%)	326	71	2.12 (1.81–7.29)
Marital status				
Ever married	513 (78.9%)	444	69	–
Unmarried	137 (21.1%)	91	46	–
Frequency of visiting health care institution (in a year)				
Never	240 (36.9%)	201	39	–
Once	130 (20%)	96	34	–
Twice	119 (18.3%)	106	13	–
Three times	96 (14.8%)	80	10	–
More than three times	65 (10%)	46	19	–
Level of healthcare service satisfaction				
Satisfied	234 (36%)	121	22	1
Averagely satisfied	114 (17.5%)	78	36	3.01 (2.17–7.25)
Not satisfied	302 (46.5%)	245	57	5.41 (2.71-14.21)

**Fig. 1:**
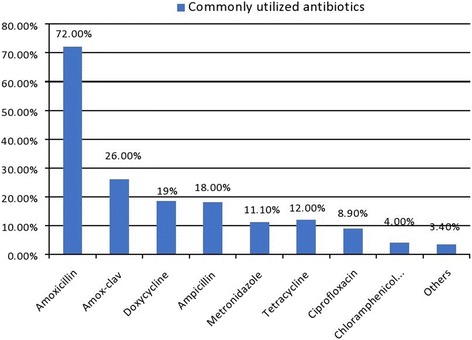
Commonly utilized antibiotics among participants

According to the finding of our study, nearly half (48.5%) of the participants took antibiotics in the past 1 year, of which 200 (63.5%) used for themselves and 115 (36.6%) used for family members. Amoxicillin (72%) was the most commonly utilized antibiotics followed by Amoxicillin-clavulanate combination (26%) and Doxycycline (19%). Commonly utilized antibiotics are depicted in Fig. 1. Respiratory tract infection (40.9%), mechanical injury/wound (27%) and acute diarrhea (19.1%) were the three most common disease conditions to which antibiotics had been taken. More than half of the respondents (53%) got antibiotics from community drug retail outlets without a prescription (36.8%) or shared from family member or neighbor (19.1%) (Table 2).

**Table 2:** Extent of inappropriate use of antibiotic among study participants, Gondar, Ethiopia 2017 (*N* = 650)

Variables	Frequency (%)
Use of antibiotics in the last 1 year	
No	335 (51.5%)
Yes	315 (48.5%)
Mode of antibiotics use	
Self-medication	200 (63.5%)
Family member medication	115 (36.6%)
Purpose of antibiotics use	
Respiratory tract symptoms	129 (40.9%)
Acute diarrhea	60 (19.1%)
Mechanical injury/wound	85 (27%)
Urinary tract symptoms	34 (10.8%)
Colic	5 (1.6%)
Headache	9 (2.9%)
I could not remember	41 (13%)
Source of antibiotics	
Prescribed by health care professional	139 (44.1%)
Bought from pharmacy without prescription	116 (36.8%)
Shared from family member or neighbor	60 (19.1%)
Discontinue treatment after symptoms subside (*N* = 315)	
No	214 (67.9%)
Yes	101 (32.1%)
Amoxicillin cure common cold	
No	125 (19.2%)
Yes	525 (80.8%)
Tetracycline cure all diseases	
No	254 (39.1%)
Yes	396 (60.9%)
Ciprofloxacin cure all types of diarrhea	
No	156 (24%)
Yes	494 (76%)

Among participants who took antibiotics, 115 (35.9%) of them used inappropriately. Majority of participants answered incorrectly on the use of amoxicillin (80.8%) tetracycline (60.9%) and ciprofloxacin (76%). Nearly two-third of respondents (67.9%) responded that they had discontinued the use of antibiotics once their symptoms gone.

### Predictors of inappropriate antibiotics use

Logistic regression analysis was employed to assess possible associations between different sociodemographic variables and respondent’ inappropriate use of antibiotics (Table 1). According to the results from bivariate logistic regression, factors that were associated with inappropriate use of antibiotics included age, educational status, employment status and satisfaction with health care services. Variables that were significantly associated with inappropriate use of antibiotics in the bivariate analysis (those with *p*-value < 0.20) were further examined in multivariate logistic regression. Having other variables controlled, educational status, employment status and satisfaction with health care services remained to be significant in the multivariate logistic model. The odds for inappropriate antibiotics use among respondents who were unable to read and write were 4.01 times higher than respondents with tertiary education. Similarly, the odds for inappropriate antibiotics use among respondents who are employed were 2.12 times higher than respondents who are unemployed. Furthermore, those who are unsatisfied with the health care services provided were 4.41 times more likely to practice inappropriate use of antibiotics than those who are satisfied with the health care services provided.

## Discussion

Inappropriate use of antibiotics, the key driver of antimicrobial resistance, is mounting at an alarming rate and the condition is conceivably worse in many developing countries including Ethiopia [23]. According to the finding of our study, 315 (48.5%) of respondents took antibiotics in the past 1 year, of which 200 (30.8%) used for themselves and 115 (17.7%) used for family members. The prevalence of self-medication reported in this study is comparable to the study done in three towns of northwest Ethiopia (27.5%) [24], but higher than studies conducted in Bahir Dar, Ethiopia (18%) [21], Portugal (19%) [25] and Euro-Mediterranean region (19.1%) [26]. Moreover, the prevalence of family medication in our study was slightly higher than the study done in Bahi Dar [21], but much lower than the study conducted in china (59.4%) [27]. Amoxicillin and Amoxicillin-clavulanate combination were the two most commonly utilized antibiotics and respiratory tract infection was the most common disease problem to which antibiotics had been sought. Similar findings were also reported in the study conducted in Uganda [28], Indonesia [29], Guatemala [30], and different parts of Ethiopia [31, 32], which reported that Amoxicillin was the most commonly used antibiotics. Similarly, in a study conducted in three selected towns of northwest Ethiopia, respiratory tract symptom was the most commonly reported complaint for self-medication [24].

It is widely believed that antimicrobial resistance can potentially arise from inadequate dosing and discontinuation of the full course of treatment [22, 33]. In our study, one third of respondents (32.1%) discontinued the use of antibiotics once their symptoms were gone. This finding is higher compared with the study done in in Kuwait (24%) [34]. But, it is lower than other studies conducted in Malaysia and Greece [35, 36]. This antibiotic misuse may put the patient at risk of relapse with drug resistant bacteria.

Several studies conducted in different parts of the globe reported that antimicrobials are purchased without a valid prescription and could be simply possessed regardless of policies prohibiting such practice [37–39]. In our study, over half of the respondents got antibiotics from community drug retail outlets without a valid prescription or shared from family member or neighbor. In a simulated study conducted in Addis Ababa, Ethiopia to assess the non-prescription sale of medications, antibiotics were obtained without any valid prescription from 75.9% of community pharmacies [40]. Similar findings were also reported in studies conducted elsewhere [41–43]. The high prevalence of non-prescription sale and access of antibiotics in our study could be due, in part, to the lack of appropriate national regulations and explicit line of actions in the sales of antimicrobials in community drug retail outlets. Furthermore, patients may prefer to go directly to pharmacies rather than visiting a hospital due to a number of reasons including ease of accessibility, shorter waiting time and accommodate patients’ ability to pay [44, 45].

According to the finding of our study, 115 (35.9%) of respondents used antibiotics inappropriately. Having other variables controlled, low educational status, being employed and unsatisfied with health care services provided are found to be strong predictors of inappropriate use of antibiotics use in the multivariate logistic model. Several other studies have also reported that lower educational status [46, 47], engagement with regular job [48, 49] and being unsatisfied with health care services [11, 12] were associated with inappropriate use of antibiotics. The low educational status of participants, which may render them to have insufficient knowledge on use of antibiotics, could have a weighty impact on the rational use of antimicrobials and development of antimicrobial resistance in the community. Hence, a customized educational campaign regarding the rational use of antibiotics and its impact on the development of antimicrobial resistance should be provided to the community. Furthermore, the relative lack of time to visit health care facilities during working hours, along with having pocket money in respondents with a regular job may render them to purchase antibiotics directly from community drug retail outlets without visiting health care facilities, ultimately increasing the potential for inappropriate use of these medications. Moreover, lack of satisfaction with health care services provided may discourage people to seek medical care from hospitals, encouraging them to look for other options for the management of their medical condition [50].

### Limitation of the study

Even though this survey highlights an area of research where there is lack of literature in Ethiopia, caution should be exercised when generalizing to other regions in Ethiopia as the study was a cross-sectional and conducted only in Gondar, northwest Ethiopia. Nevertheless, this survey has significant implications for promoting the rational use of antibiotics and contain the development of antimicrobial resistance in the community.

## Conclusion

The results of the present study revealed that inappropriate use of antibiotics is high and associated with low educational status, engagement in regular job and being unsatisfied with health care services. Our findings emphasize the need to form and deliver a comprehensive and multifaceted interventions including providing a customized education to the public so as to improve their knowledge about antibiotics and change their attitude to limit self-medication. Moreover, it is essential to adopt a strong and explicit line of actions towards the accessibility of antibiotics without a valid prescription. A larger national scale and multi centered survey that includes more diverse participants is warranted to validate our findings and to provide more accurate findings. In addition, we do recommend upcoming studies to focus on identification of areas to antibiotic treatment failure and referral to healthcare facilities due to infectious diseases like tuberculosis.

## Abbreviations

AMR: Antimicrobial resistance
AOR: Adjusted odds ratio
CI: Confidence interval
FMHACA: Food, medicine and healthcare administration and control Authority of Ethiopia
MDR: Multi-drug resistant
OR: Odds ratio
SPSS: Statistical package for the social sciences
USD: United States Dollar
WHO: World Health Organization
